# Agriculture-Induced N_2_O Emissions and Reduction Strategies in China

**DOI:** 10.3390/ijerph191912193

**Published:** 2022-09-26

**Authors:** Guofeng Wang, Pu Liu, Jinmiao Hu, Fan Zhang

**Affiliations:** 1 Faculty of International Trade, Shanxi University of Finance and Economics, Taiyuan 030006, China; 2 Think Tank for Eco-Civilization, Chinese Academy of Social Sciences, Beijing 102445, China; 3 Key Laboratory of Land Surface Pattern and Simulation, Institute of Geographical Sciences and Natural Resources Research, Chinese Academy of Sciences, Beijing 100101, China

**Keywords:** agricultural N_2_O emissions, emission intensity, spatial analysis, reduction strategies

## Abstract

Greenhouse gases are one of the most important factors in climate change, their emissions reduction is a global problem. Clarifying the spatial patterns of N_2_O, as an important component of greenhouse gases, it is of great significance. Based on the planting and breeding data of China from 2000 to 2019, this paper measures the N_2_O emissions of agricultural systems, and uses kernel density to explore the spatial distribution differences between the eight major economic zones. Finally, the proposed emissions reduction countermeasures are provided. The research results show that the N_2_O emissions of China’s agricultural system showed a trend of increasing first and then decreasing, and in 2019, the national N_2_O emissions were 710,300 tons, agricultural land emissions and animal husbandry emissions were the main sources of N_2_O emissions. The difference in N_2_O emissions by province was significant, the concentration trend was more prominent, and the differences of N_2_O emissions between provinces and regions were diverse. In order to achieve the reduction in N_2_O emissions, it is necessary to carry out low-carbon production of staple grains for different parts and economic zones, and focusing on low-carbon production in the Central Part and the West Part, as well as the Northeast and the Greater Southwest zones, is essential.

## 1. Introduction

Global warming and ozone destruction are the two major environmental problems plaguing human development [1]. Global warming not only has an negative impact on the environment, water resources, regional sea level rise, agriculture, natural vegetation, etc. [2], but also influences human health [3]; the prolongation of hot summer weather and high temperature and high humidity weather directly threaten people in the mid-latitude regions that are sensitive to the warming of the earth. Moreover, global warming provides a better hotbed for the reproduction and spread of many pathogens, which will make many infectious diseases spread from the tropics and subtropics to the north and south, thus the threatened population will increase, and the epidemic time will be extended [4]. How to mitigate and adapt to climate change has become an important challenge for countries around the world.

Greenhouse gases are one of the important causes of climate change, and how to reduce N_2_O emissions has become a common problem faced by all mankind [5]. In the Kyoto Protocol, N_2_O is designated as one of the greenhouse gases [6]; it is low in the atmosphere, but due to the huge destructiveness of its individual molecules in terms of warming, the greenhouse effect it can cause is 268 times that of CO_2_ [7]. Thus, it is the third largest greenhouse gases after CO_2_ and methane [8], and with increased global population and nitrogen fertilizer use, N_2_O emissions have increased dramatically [9]. The increase in atmospheric N_2_O concentrations over the past 150 years has contributed to stratospheric ozone depletion and climate change [10], and after N_2_O enters the atmosphere, it needs 120 years to fully decompose [11]. Its future net contribution to the warming from 2010 to 2100 is about 0.1 °C (50% probability) [1]. Furthermore, N_2_O is a major component of nitrogen oxides—a leading cause of premature death in humans and the decline in biodiversity worldwide [5]. The N_2_O produced by human activities mainly comes from agricultural production, and the N_2_O emitted by agricultural soil accounts for 52% of the N_2_O emitted by human activities [12]. The main ways of N_2_O production in farmland soil are nitrification process and denitrification process, and denitrification is considered as the most important process of N_2_O emission in farmlands. In addition, in agricultural soil, the application of nitrogen fertilizer is an important driving factor for N_2_O production, and the excessive application of nitrogen fertilizer (the amount of nitrogen fertilizer applied exceeds the part absorbed by crops in the current season) is an important reason for N_2_O emission in farmland soil [13].

China’s agricultural development is gradually transforming towards greening. The development of global agriculture has entered the process of refined and green development [14], and China is no exception. Since 2015, the Ministry of Agriculture and Rural Affairs has organized a zero-growth action for the use of chemical fertilizers and pesticides, and after 5 years of implementation, by the end of 2020, China’s chemical fertilizer and pesticide reduction and efficiency have successfully achieved the expected goals, the use of chemical fertilizers and pesticides has significantly reduced, and the utilization rate of chemical fertilizers and pesticides has significantly improved [15]. The high-quality development of the planting industry has achieved obvious results. In 2020, the fertilizer utilization rate of China’s three major grain crops of rice, wheat and corn was 40.2%, with an increase of 5% over 2015; the utilization rate of pesticides was 40.6%, with an increase of 4% over 2015. In 2020, the national organic fertilizer application area exceeded 550 million mu, and the proportion of high-efficiency and low-risk pesticides exceeded 90%. China’s N_2_O emissions have the characteristics of regional concentration [16], and their concentration characteristics and evolution need to be clarified urgently. Due to the difference in climatic conditions, there are large differences in the cultivation of staple grain crops in the north and south of China. In addition, there are huge differences in the species and number of farmed species. To this end, clarifying regional differences, identifying the sources of differences, and exploring regional cooperative emission reduction programs are of great significance for climate change.

Estimating and clarifying the sources of N_2_O emissions from agricultural systems has always been a hot topic in academic research of China and abroad [17,18,19,20]. Using top- bottom and bottom-top methods to measure N_2_O emissions from agricultural systems is reasonably consistent across the globe. Dietary demand affects planting and animal husbandry, and modeling of population and dietary patterns, are critical for measuring future emission fluxes [21]. Manure storage and manure spread in different environments, examined soil types, fertilization types, fertilizer amounts, and the impact of mud treatment, manure management affects direct and indirect N_2_O emissions in each country and that DM content, and manure and soil temperature are key to farm manure management [20]. Based on three-year field measurements, N_2_O emissions were triggered by mid-season drainage compared to persistent flooding and were mainly dependent on stagnant water and indirect irrigation. Meanwhile, straw returning had a significant effect on N_2_O, but decreased slightly, and N_2_O emissions induced by crop residues and fertilizers depended on the moisture status of rice [22]. Stabilizing climate change below 2 °C and close to 1.5 °C requires mitigation of all greenhouse gases, including CO_2_ and non-CO_2_ emissions, based on the GLAM model, the impact of non-CO_2_ gases on mitigating climate pressures is reflected in the 2 °C pathway reaching net zero CO_2_ emissions linearly in five-year increments between 2030 and 2100, followed by zero CO_2_ emissions. The 1.5 °C pathway reaches −8 Gt CO_2_ yr^−1^ CO_2_ linearly in five-year increments be-tween 2030 and 2100, followed by emissions after −8 Gt CO_2_ yr^−1^ CO_2_. In the future, the reduction in non-CO_2_ emissions will depend on the level of CO_2_ emissions reduction, as well as the non-CO_2_ emissions reduction measures; CO_2_ emissions must reach net zero two decades ago to achieve the same climate goals without specific non-CO_2_ emissions [23]. N_2_O emissions increase exponentially when nitrogen use exceeds optimal levels. In addition, existing empirical models of N_2_O response to N rate overestimate N_2_O emission in the North China Plain to some extent, even at high N rate [24]. As livestock production in China is increasingly located near urban areas, human populations are exposed to nitrogen pollution via air and water. So, China may have to relocate 5–10 billion animals to tackle nitrogen pollution of air and water caused by concentrations of livestock production. Relocating animals from eastern and southern China is challenging and has huge economic and social implications [15]. We should be aware that meteorology, soil parameters, and practice managements could substantially influence soil N_2_O emissions; applying nitrification inhibitor and reduced N fertilizer (20%) could decrease 62% and 10% of soil N_2_O emissions, respectively [25]. Substantial policy interventions are an important guarantee of achieving the goal set out in the 2016 Paris Agreement to maintain the global temperature rise below 2 °C this century, or even 1.5 °C above pre-industrial levels, especially for industrialized countries [2]. The research on N_2_O in agricultural systems is not perfect, especially the overall differences in spatial emissions. This paper measures the N_2_O emissions from agricultural systems in 31 provinces, municipalities, and autonomous regions (excluding Hong Kong, Macao and Taiwan) in China from 2000 to 2019; the overall emission, regional emission and the spatial distribution provincial emission of N_2_O emissions in China from 2000 to 2019 are calculated, the differences and changes are analyzed. As a traditional agricultural country, China has typical characteristics of agricultural N_2_O emission. The study on agricultural N_2_O emission in China will provide reference for the world’s agricultural N_2_O emission reduction.

## 2. Materials and Methods

### 2.1. Method for Estimating N_2_O Emissions

The N_2_O emissions of the national agricultural system are mainly from the direct emissions of agricultural land for planting and the emissions from livestock and poultry manure management in animal husbandry industry [26], and the formula is calculated as:

(1) $$E_{N_{2}O}=\sum (E_{N_{2}O_{direct}}+E_{N_{2}O_{manure}})$$

Among them, the direct emissions from the planting industry source from the N_2_O produced by the amount of fertilizer applied on agricultural land and the return of straw to the field, and the formula is calculated as:

(2) $$E_{N_{2}O_{direct}}=(E_{N_{fertilizer}}{+E}_{N_{straw}})\times EF_{direct}$$

In the formula, $E_{N_{2}O_{direct}}$ is the direct emission of N_2_O from agricultural land for planting industry, ×10^4^ t; $E_{N_{fertilizer}}$ is the amount of chemical fertilizer applied for agricultural land, ×10^4^ t; $E_{N_{straw}}$ is the nitrogen returned to the field of straw, ×10^4^ t; $EF_{direct}$ is the direct emission factor of N_2_O for agricultural land (Table 1).

The nitrogen produced by straw returning to the field is calculated as follows:

(3) $$E_{N_{straw}}=\sum_{i=1}^{n}M_{i}/L_{i}-M_{i}\times \beta_{i}\times K_{i}{+M}_{i}/L_{i}\times \alpha_{i}\times K_{i}$$

In the formula, $E_{N_{straw}}$ is the nitrogen of straw return, ×10^4^ t; $M_{i}$ is the grain yield of crop i, ×10^4^ t; $L_{i}$ is the economic coefficient of crop i; $\beta_{i}$ is the straw return rate of crop i; $K_{i}$ is the straw nitrogen content of crop i; $\alpha_{i}$ is the root-to-crown ratios of crop (Table 2).

The formula for calculating N_2_O generated by manure management in animal husbandry is calculated as follows.

(4) $$E_{N_{2}O_{manure}}=\sum_{i=1}^{n}EF_{N_{2}O_{manure}}\times AP_{i}\times 10^{-7}$$

In the formula, $E_{N_{2}O_{manure}}$ is N_2_O emissions of livestock manure management, ×10^4^ t; $EF_{N_{2}O_{manure}}$ is the emission factor for livestock and poultry manure management of category i, $AP_{i}$ is the number of livestock and poultry of category (Table 3).

### 2.2. Method for Estimating Kernel Density

Kernel density estimation is a nonparametric test that uses a continuous density function curve to describe the distribution pattern of a random variable. In this paper, the nu-clear density estimation curve is used to depict the temporal evolution characteristics and differences of N_2_O emissions from agricultural systems in eight major economic zones of China, and the formula is:

(5) $$f\left(x\right)=\frac{1}{Nh}\sum_{i=1}^{N}K\left(\frac{X_{i}-x}{h}\right)$$

Among them, $X_{i}$ represents N_2_O emissions from agricultural systems in each province. x is the mean value of N_2_O emissions from the agricultural system, N is the total sample number, h is the bandwidth, and K is the Kernel function.

### 2.3. Data Sources and Regional Segregation

#### 2.3.1. Data Sources

The crop yield and livestock and poultry number in this paper are derived from China Statistical Yearbook (2000–2019), State Administration of Grain, and China Agricultural Yearbook (2000–2019). The emission factors of N_2_O on agricultural land in different regions, the main crop parameters and the emission factors of livestock and poultry manure management in different regions are all from the Guidelines for the Preparation of Provincial Greenhouse Gas Clean-up (National Development and Reform Commission, 2011). Below is some indicator information (Table 4).

#### 2.3.2. Regional Segregation

**Figure 1:**
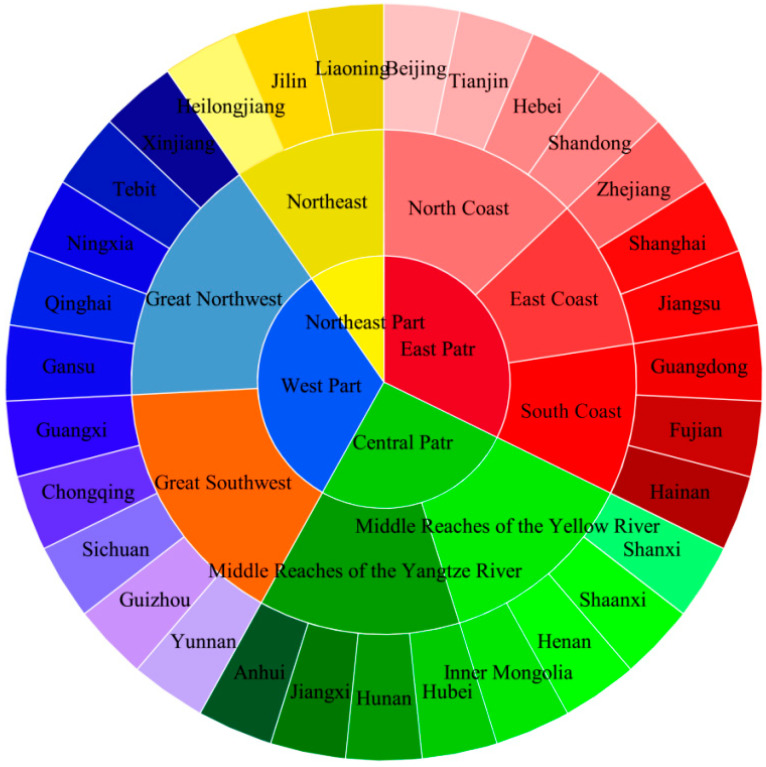
Regional segregation of agricultural N_2_O emissions in China.

In order to study the differences and internal correlations between different regions, this study adopts the division method of four major parts and eight major economic zones, of which four major parts include the East, Central, West and Northeast, and the eight major economic zones contain the North Coast, East Coast, South Coast, Middle Reaches of the Yellow River, Middle Reaches of the Yangtze River, Great Southwest, Great North-west and Northeast (Figure 1)

## 3. Results

### 3.1. Overall Analysis of N_2_O Emissions

**Figure 2:**
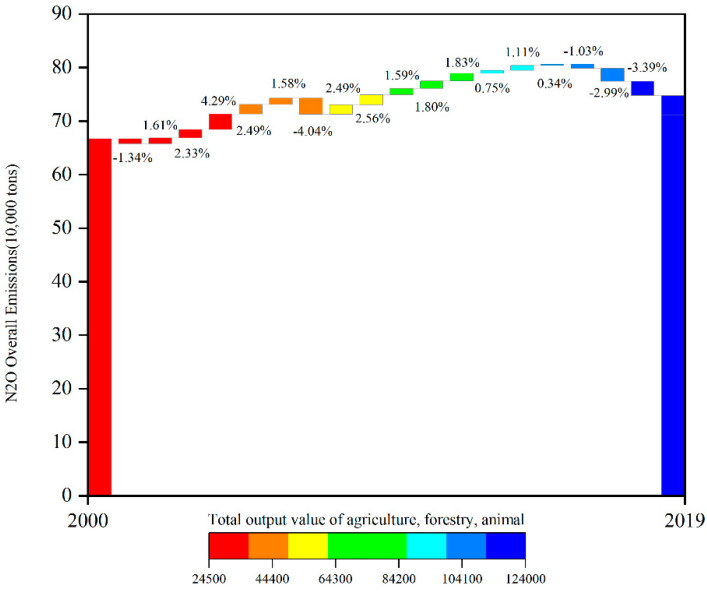
Agriculture-induced N_2_O Emissions from 2000–2019 in China.

China’s N_2_O emissions showed a trend of rising first and then falling (Figure 2). In 2019, the N_2_O emissions of agricultural system in China were 710,300 tons, down 5.03% year-on-year, of which N_2_O emissions generated by chemical fertilizer application were 514,100 tons, produced by straw returning to the field were 0.05 million tons, and formed from livestock and poultry manure management were 195,700 tons, accounting for 72.38%, 0.07% and 27.55%, respectively. Between 2000 and 2019, the N_2_O emissions of the agricultural system increased from 666,700 tons to 710,300 tons, with an average annual growth rate of 0.33%. In terms of total amount, agricultural N_2_O emissions increased, but experienced a trend of first rising and then declining. To be specific, 2000–2015 was the uptrend period, the agricultural N_2_O emissions increased from 666,700 tons to 806,300 tons, with an annual growth rate of 1.28%, while 2007 was a special year, with a 4.03% decrease compared with 2006. 2016–2019 was the downtrend period, with the agricultural N_2_O emissions dropping from 798,000 tons to 710,300 tons. The green level of agricultural production has increased since 2016, and it can be inferred from the trend that agricultural N_2_O emissions will continue to decline in the next few years.

From the perspective of three main sources of agricultural N_2_O emissions (Agricultural land fertilizer, Straw return, livestock, and poultry manure management), agricultural land N_2_O emissions increased as a whole, with 401,900 tons in 2000 and 514,100 tons in 2019, which was mainly due to the increased use in the application of chemical fertilizers on agricultural land. It indicates that the increase in China’s agricultural output depended on the input of agricultural data to some extent; animal husbandry N_2_O emissions decreased from 264,500 tons in 2000 to 195,700 tons, which was because of the reduction in livestock and poultry number and the change in breeding structure. Straw return N_2_O emissions increased from 0.03 million tons to 0.05 million tons. In terms of proportion, the percentage of emissions from chemical fertilizer application increased from 60.28% to 72.38%, the proportion of animal husbandry decreased from 39.67% to 27.55%, and the share of straw return emissions rose from 0.04% to 0.07%. The year of 2007 had the biggest change, compared with 2006, the proportion of emissions from animal husbandry decreased from 36.03% to 31.04%, and the percentage of emissions from fertilizer application increased from 63.93% to 68.90%, with a change range of nearly 5%. This change was due to the support of strengthening agriculture, rural areas and farmers, developing modern agriculture and promoting the construction of new countryside in No. 1 Central Document of 2007.

### 3.2. Provincial Distribution of N_2_O Emissions

**Figure 3:**
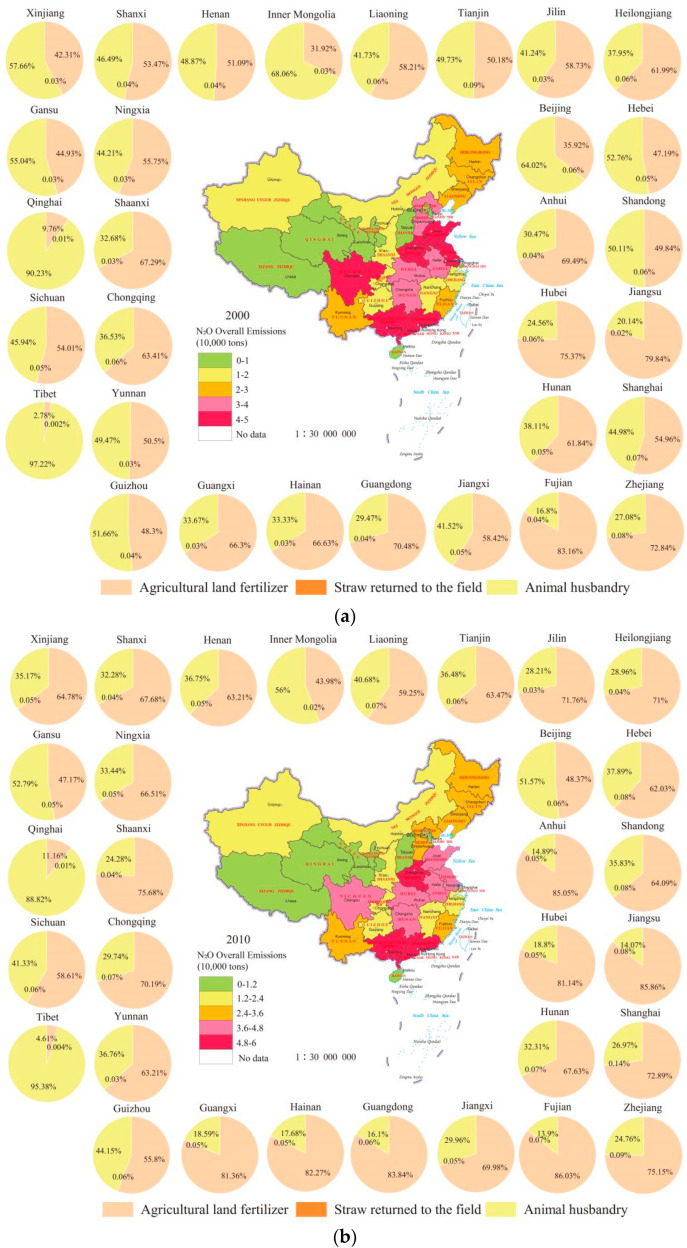

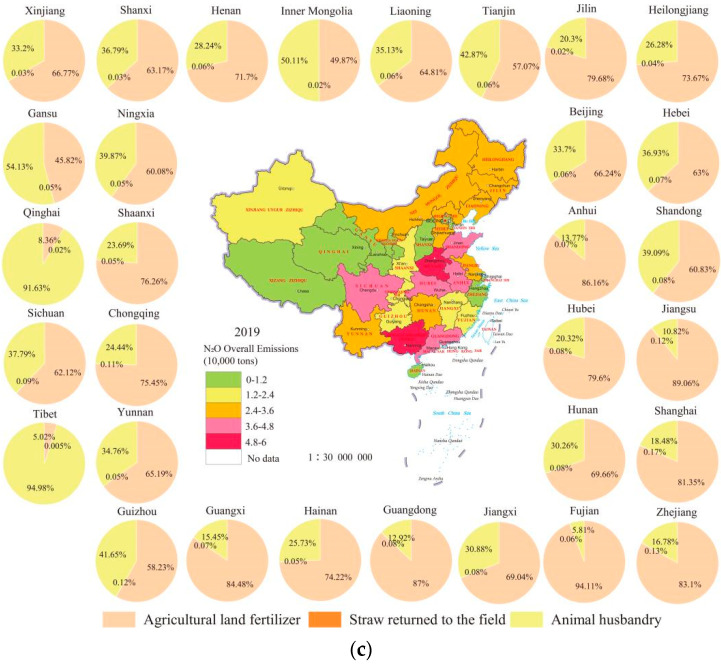
Provincial N_2_O Emissions from 2000–2019 in China ((**a**) 2000; (**b**) 2010; (**c**) 2019).

In terms of the overall characteristics of each province, the overall differences be-tween different provinces, municipalities and autonomous regions are large (Figure 3). In 2019, the smallest N_2_O emissions were in Beijing, at 0.053 million tons, and Guangxi, which had the largest emissions, reached 5.3 The main difference is that the amount of agricultural land in Beijing and Guangxi is different, and the emission of N_2_O from agricultural land in Guangxi in that year was 4.49 million t. From 2000 to 2019, the provinces with reduced N_2_O emissions were mainly distributed in provinces with relatively rapid urbanization, such as Beijing, Shanghai, Tianjin, etc., which was directly related to the reduction in agricultural land in these areas. In particular, Beijing’s N_2_O emissions have dropped from 0.28 million tons to 0.053 million tons, down 81.07%.

N_2_O emissions in various provinces showed three kinds of changes, the main forms of which were significant decline, significant increase and stable change. Among them, Beijing, Tianjin, Shanghai, Hebei and Jiangsu have declined significantly, and these areas have rapidly urbanized between 2000 and 2019, and the urbanization rate in 2019 has reached 86.6%, 83.48%, 88.3%, 57.42%, 70.4%, respectively. Ningxia, Gansu, Shaanxi, Xin-jiang, Inner Mongolia and other underdeveloped areas showed a significant upward trend in N_2_O. The N_2_O emissions of Shandong showed a downward trend, and the N_2_O emissions increased from 4 in 2000. 84 million tons fell to 37,000 tons in 2019, of which, planting N_2_O emissions fell from 24,100 tons to 22,600 tons N_2_O emissions from aquaculture decreased from 24,300 tons to 14,500 tons, the possible reason for the decline in emissions is the shift from local crop cultivation to vegetable cultivation, coupled with the adjustment of the number and structure of culture.

Provinces with more N_2_O emissions are more concentrated, mainly concentrated in Guangxi, Henan, Jiangsu and other provinces, and in 2019, N_2_O emissions were respectively, 53,100 t, 53,000 t, 35,000 t, The common feature of these provinces is that they belong to large planting provinces, taking Henan Province as an example, its planting industry N_2_O emissions are 38,000 tons, and the aquaculture industry emissions are 1 490,000 tons, therefore, it is of great significance to do a good job in reducing N_2_O emissions in the planting industry.

### 3.3. Regional Variation of Agricultural N_2_O Emissions

**Figure 4:**
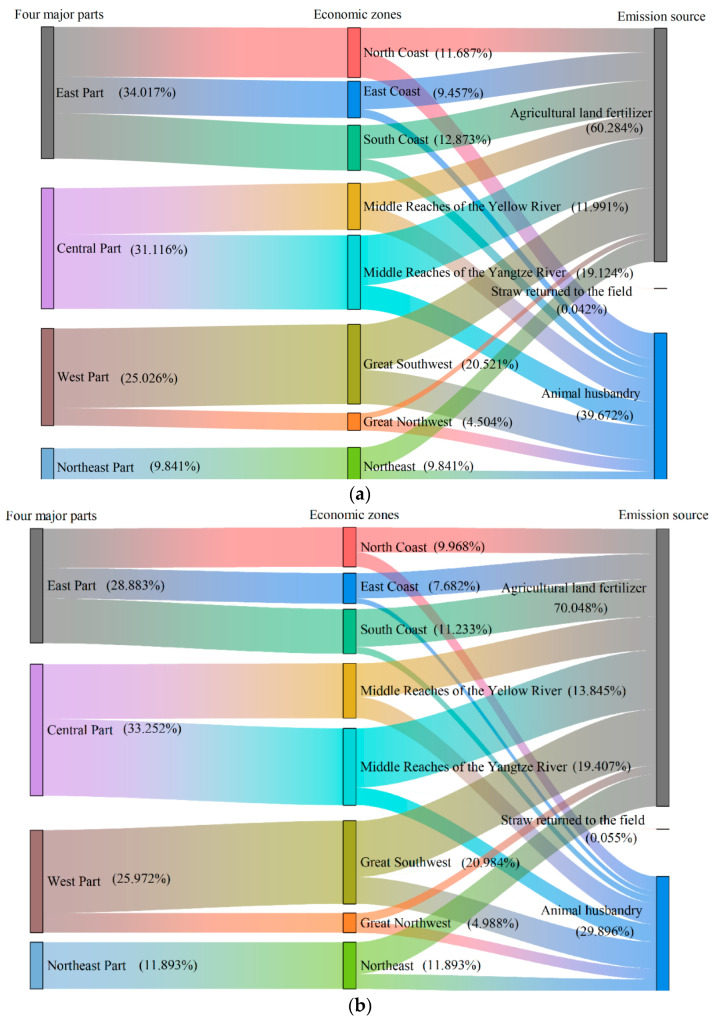

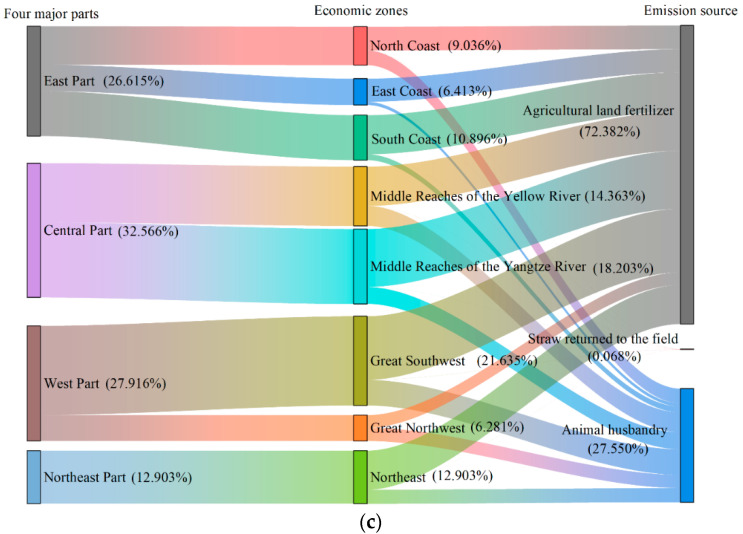
Agriculture-induced N_2_O emissions of economic zones from 2000 to 2019 in China ((**a**) 2000; (**b**) 2010; (**c**) 2019).

In terms of total emissions amount, from the perspective of four major parts (Figure 4), the largest amount of agricultural N_2_O emissions was the Central, because it is the main grain and livestock production area in China. It was followed by the East, and distinct from other parts, this part showed a downward trend, indicating that agricultural technology has been promoted on a large scale and agricultural production has become greener. Relying on the developed economic level in the eastern region, the effect of agricultural science and technology progress is better. Compared with the early stage of economic development, some low-carbon agricultural technologies and development models have been explored and promoted in practice, such as the precision agriculture input model based on soil testing, formula fertilization and water-saving irrigation, and the agricultural waste treatment and utilization model based on manure management, straw recycling and household biogas. Through promoting the integration of water and fertilizer, replacing chemical fertilizer with organic or green fertilizer, and other measures, such as nitrogen efficiency enhancement or organic substitution, to reduce the amount of application and inhibit N_2_O emission. The N_2_O emissions of the West and the Northeast ranked third and fourth, respectively, their N_2_O emissions were on the rise before 2015 and declined significantly after 2015, with 198,300 tons and 91,600 tons, respectively, in 2019.

N_2_O emissions from each part showed the characteristics of regional concentration. Except for the East, other parts all showed an increasing trend, the Central, West and Northeast Part increased by 11.52%, 18.84% and 39.63%, respectively, and the main reason for this change was the promotion of urbanization on the occupation of agricultural land. From the perspective of four major parts, the part with the largest N_2_O emissions was the Central, which was mainly concentrated in the Middle Reaches of the Yellow River and the Middle Reaches of the Yangtze River. In 2019, the N_2_O emissions of Central were 231,300 tons, which was closely related to the fact that the Central is the main producing area of grain and animal husbandry in China. The second was the East, which was quite different from the national evolution trend, and the N_2_O emissions of this part peaked at 232,300 tons in 2005, after which, the N_2_O emissions of the East fluctuated downwards. The N_2_O emissions of the West and the Northeast ranked third and fourth, and the North-east has the reputation of “China’s granary”. The N_2_O emissions of these two parts dropped significantly after 2015, which may be related to the expansion of the mechanization of farming and the integration of green development methods in these regions.

From the perspective of the eight major economic zones (Figure 4), the top three regions in terms of emissions amount were the Great Southwest, the Middle Reaches of the Yangtze River, and the Middle Reaches of the Yellow River, which was mainly due to climate, water, crops and other reasons; importantly, these zones are more suitable for the development of animal husbandry and planting. In addition, affected by the technical level and farming methods, the level of green production was limited, thus the emissions amount in these areas were relatively high. The Great Northwest had the least emissions amount, and in 2000, the N_2_O emissions amount in the Great Southwest were 4.6 times that of the Great Northwest, then dropped to 3.4 times in 2019. The regional differences in agricultural N_2_O emissions were gradually narrowing.

The main sources of changes in agricultural N_2_O emissions are chemical fertilizer ap-plication, straw return, and livestock and poultry manure management. Among them, the areas where N_2_O emissions from agricultural systems change mainly due to livestock and poultry manure management were the East Part and the North Coast. In other areas, N_2_O emissions in agricultural system was changed all due to chemical fertilizer application in agricultural land.

Distinct from other parts, the East Part showed a downward trend, from 226,800 tons in 2000 to 189,000 tons in 2019, with a decrease of about 16.65%, and the downward momentum was mainly driven by the decline in N_2_O emissions from the animal husbandry caused by the reduction in the number of livestock and poultry, which showed that the industrial structure and feeding structure have been optimized; the Central Part has increased by 23,900 tons, with an increase of about 11.5% between 2000 and 2019; the West Part had the largest increase amount, about 31,400 tons, growing by 18.84%; the Northeast Part had the largest growth rate, about 39.69%, growing by 26,000 tons. In addition, the animal husbandry in three parts N_2_O emissions have all declined. The main reason for the increase in agricultural N_2_O emissions was the increase in planting N_2_O emissions, indicating that planting emission reduction is of great significance for the realization of dual carbon goals. Similar to the four major parts, the three economic zones including the North Coast, the East Coast and the South Coast of the East Part all showed an overall downward trend, with a decline of 22.99%, 27.76% and 0.67%, respectively. Other regions which also showed an upward trend and had the largest increase rate was the Great Northwest, with about 48.54%. As its total emissions were the least, it only increased by 14,600 tons, while Northeast saw the biggest increase by 26,000 tons. From the provincial level, on the one hand, the provinces with largest change rate were Xinjiang, Inner Mongolia, Beijing and Shanghai, and the change rate was 106.34%, 87.02%, −81.31%, −73.64%, respectively. On the other hand, the provinces with largest increase amount were Heilongjiang, Inner Mon-golia, Xinjiang, Guangxi, and Jilin, all with an increase amount of more than 10,000 tons. Besides, Provinces with more N_2_O emissions were more concentrated, mainly gathering in Guangxi, Henan, Jiangsu and other provinces and most of these provinces are large planting provinces.

### 3.4. Kernel Density of Eight Major Economic Zones

**Figure 5:**
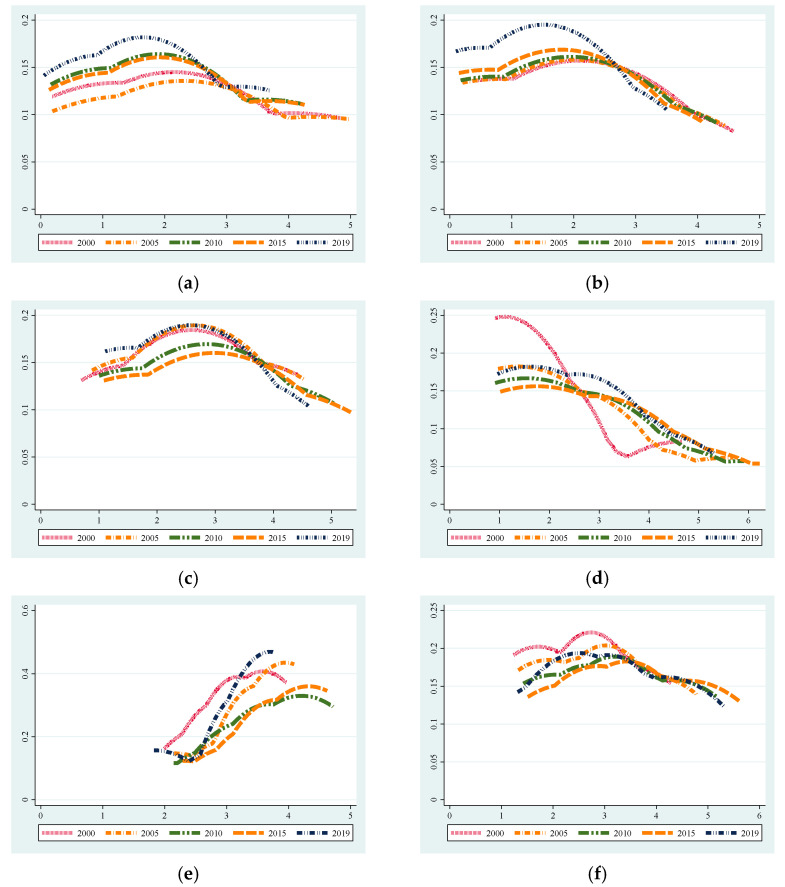

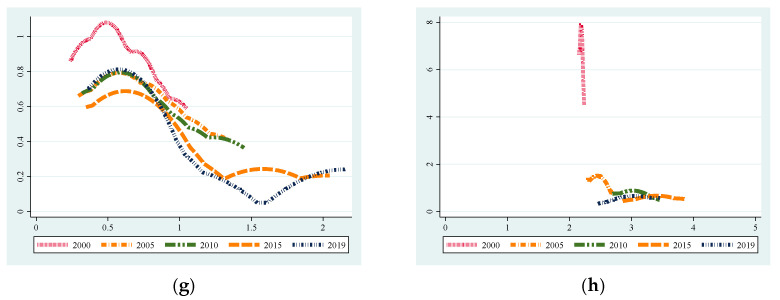
Nucleus density curves of agriculture induced N_2_O emissions in economic zones (**a**) North Coast economic zones (**b**) East Coast economic zones (**c**) South Coast economic zones (**d**) Middle Reaches of the Yellow River economic zones (**e**) Middle Reaches of the Yangtze River economic zones (**f**) Great Southwest economic zones (**g**) Great Northwest economic zones (**h**) Northeast economic zones.

In order to effectively reflect the spatial differences of agricultural N_2_O emissions, the dynamic distribution of the five time periods of 2000, 2005, 2010, 2015 and 2019 in the eight major economic zones was analyzed by using non-parametric kernel density (Figure 5). The shape of the nuclear density curve in the North Coast and the East Coast was single-peaked, of which the peak in the North Coast shifted to the left, indicating that the emissions were reduced, and the East Coast shifted to the left with the passage of time, reflecting the gradual narrowing of the differences between the provinces within the zones; the peak position of South Coast were similar in 2019 and 2000, and the overall emissions did not change much; the peak shape of the Middle Reaches of the Yellow River, the Middle Reaches of the Yangtze River, the Great Southwest and the Great Northwest were multi-peaked or had a multi-peak trend, indicating that there was a multi-level differentiation phenomenon. In addition, the Middle Reaches of the Yellow River trailed to the right, and the internal differences became larger. The curve of the Middle Reaches of the Yangtze River first shifted to the right, and then shifted to the left in 2019, indicating that the inter-provincial emission difference had an expanding trend from 2000 to 2015, but it showed a decreasing trend from 2015 to 2019. The relative change interval of the Great Southwest and the Great Northwest widened, and the internal difference increased; the Northeast was special, it was steeper in 2000, and then evolved into a flat shape, indicating that inter-provincial differences were gradually widening.

## 4. Conclusions and Discussion

### 4.1. Conslusions

This paper calculates the N_2_O emissions generated by the main emission sources of agricultural systems (fertilizer application, straw return, livestock and poultry manure management) in different provinces of China from 2000 to 2019, introduces nuclear density analysis, and divides China into four major parts and eight major economic zones to study the temporal and spatial patterns of N_2_O emissions from agricultural systems. The specific conclusions are as follows:

Firstly, overall changes in N_2_O emissions from agricultural systems. Between 2000 and 2019, the overall trend of N_2_O emissions from the agricultural system was initially increasing and then declining, of which 2000–2015 was the rising period, after 2016 was the decline period, and the national agricultural N_2_O emissions in 2019 were 710,300 tons. Among them, the N_2_O emissions generated by the application of chemical fertilizers were 514,100 tons, produced by the livestock and poultry manure management were 195,700 tons, formed from straw returning to the field were 0.05 million tons. The emission intensity dropped from 0.268 (ton/million yuan) to 0.057 (ton/million yuan). From the changing trend, agricultural N_2_O emissions amount and emission intensity will continue to decline, and agricultural production will be more efficient.

Secondly, regional changes in N_2_O emissions from agricultural systems. From the perspective of regions, the top three economic zones in terms of the emissions amount were the Great Southwest, the Middle Reaches of the Yangtze River, and the Middle Reaches of the Yellow River. The Great Southwest had the highest N_2_O emissions intensity, followed by the Great Northwest, the Northeast and the Middle Reaches of the Yangtze River. The regional differences in emissions amount and emission intensity were gradually narrowing, and the Great Southwest is the significant N_2_O emission reduction zones.

Thirdly, changes in differences. The difference between regions of N_2_O emissions amount and the N_2_O emission intensity were gradually narrowing, and the differences in the North Coast, the East Coast and the Middle Reaches of the Yangtze River also had a clear trend of narrowing. 2007 was a key year for the reduction in N_2_O emissions in the agricultural system, whether it was the total amount of N_2_O emissions or the intensity of N_2_O emissions, the range of changes was obvious, which was a turning point for China’s agricultural N_2_O emission reduction and the realization of the dual carbon goal. In addition, the increase in N_2_O emissions in different regions was caused by agricultural chemical fertilizers, so it is of great significance to focus on the emissions reduction in planting industry.

### 4.2. Disscussion

Agriculture is an important source of global N_2_O emissions, and global anthropogenic N_2_O emissions have increased substantially. The average concentration of N_2_O in the atmosphere increased to 331 μg·L^−1^ in 2018, and the continuous increase in agricultural emissions was an important driving factor, accounting for more than 60% of global anthropogenic N_2_O emissions. Therefore, actively promoting agricultural N_2_O emission reduction is of great significance to mitigate global climate change. According to data released by the World Bank, global agricultural N_2_O emissions in 2019 were equivalent to 2,298,470 thousand metric tons of carbon dioxide. As a traditional agricultural power, China’s N_2_O emissions from agriculture in 2019 were equivalent to 342,820 thousand metric tons of carbon dioxide, accounting for a high proportion in the world. India, which is also an agricultural powerhouse in Asia, with 72% of its population living in rural areas, emitted equivalent 222,200 thousand metric tons of carbon dioxide from agriculture in 2019. In 2019, N_2_O emissions from agricultural systems in the United States, which has developed agriculture and a high degree of mechanization, were equivalent to 181,600 thousand metric tons of carbon dioxide. As the largest developing country and a major agricultural country, China should focus on reducing agricultural emissions and developing low-carbon agriculture.

From the estimation results, it is found that the development of low-carbon agriculture in China should focus on the central and western regions. The central region is the concentrated area of China’s planting and breeding industries, including the Yellow River Basin and the Yangtze River Basin. This region has flat terrain and suitable climate. As a major grain-producing area, the intensity of crop production activities is high, and the pressure on agricultural N_2_O emission reduction is also great. At the same time, the central provinces are undertaking a large number of location transfers of animal husbandry in the east and are under great pressure in terms of infrastructure and green development. The western region is a traditional animal husbandry area in China. It has a vast area but fragile ecology. Animal husbandry production is mainly based on grazing. It faces a series of problems such as overload grazing and grassland degradation. The manure treatment technology needs to be improved. It is also under pressure to reduce agricultural N_2_O emissions. Compared with other regions, the eastern region is economically developed. The technology level in this region is relatively high, and it is easy to achieve a win-win situation of agricultural economic and ecological benefits. At the same time, the high urbanization rate is conducive to promoting the scale and intensification of agricultural production, improving labor productivity and green productivity. On the other hand, rapid urbanization is conducive to promoting the adjustment of the agricultural industry structure, and puts forward higher requirements for technological innovation, the development of low-carbon agricultural technology, and the high-quality development of agriculture. Among the eight economic zones, the Great Southwest had the largest agricultural N_2_O emission, indicating that while pursuing economic development, strengthening the promotion and use of low-carbon agricultural technologies is an important measure that needs urgent attention in this region.

Considering the originality and limitations, this paper not only calculated the total N_2_O emissions of China’s agricultural system from 2000 to 2019, but also estimated the emissions of different regions in the four major parts and eight economic zones, as well as their differences. However, there are also limitations. Firstly, due to data limitations, there is a lack of estimation of N_2_O indirect emissions from agricultural systems. Secondly, this paper only estimated the situation in China, so future research can focus on other countries and the comparison among different countries.

## 5. Suggestions

Based on the existing research results and the actual situation of Chinese agriculture, the following aspects can be considered for curbing N_2_O emission from Chinese agriculture.

Firstly, nitrogen use efficiency can be optimized at the median nitrogen input by postponing the timing of fertilization. Production efficiency can be improved through scientific farming, the use of inhibitors, and the adjustment of agricultural industrial structure. In addition, through the substitution of organic fertilizer, the resource utilization system of livestock and poultry breeding waste can be improved, the green planting and breeding cycle can be promoted, and the comprehensive utilization of straw can be strengthened. The N_2_O emission can be reduced by adjusting the manure storage environment, improving the manure treatment method, and adopting anaerobic conditions. It is also possible to explore ways of combining planting and breeding to control N_2_O emissions.

Secondly, for the four major agricultural lands and areas with more animal husbandry, mainly in the central and western regions, the efficiency of planting and breeding can be improved by improving production technology and large-scale planting. For example, the proper use of shallow water irrigation and the implementation of conservation tillage on the basis of reasonable allocation can effectively reduce N_2_O. At the same time, proper adjustment of the breeding structure is an effective low-carbon measure.

Thirdly, for regions with large internal differences, spatial cooperation can be carried out, technologies and green production models can be promoted, and agricultural circular production and industrial circular combination can be implemented to promote efficient use of N_2_O, reduce emissions, and ultimately achieve low-carbon emissions.

Fourthly, it is necessary to focus on areas with large internal differences, such as the Northeast and the Great Southwest of the eight major economic zones. The combination of agricultural circular production and industrial circulation should be promoted and the efficient use of N_2_O should be encouraged to reduce emissions in order to achieve low-carbon production of major food crops. In addition, reducing crude protein (CP) content in animal feed rations, increasing the use of pulp acidification, thermal drying, incineration and pyrolysis, separating and processing pulp, and converting manure management from a liquid system to a solid-liquid separation system are management strategies.

In conclusion, the measures focusing on agricultural N_2_O emissions reduction should not only consider natural conditions such as soil, climate, and water, but also comprehensively consider agricultural technology, nitrogen fertilizer use, farming methods, industrial structure and other factors. Agricultural nitrogen pollution is related to groundwater quality, human and ecosystem security, we need to develop methodologies to quantify nitrogen legacies and lag times. This could include, for example, quantifying lag times and adjusting expectations; using legacy as a resource; spatial targeting of watershed conservation measures; couple field-scale and downstream measures to minimize lag times; diversify monitoring to evaluate outcomes and inform adaptive management; better incorporate assessments of both long-term and short-term benefits into economic analyses. In addition, while conducting research on emission reduction measures according to the situation of each region, the emissions of other greenhouse gases caused by anthropogenic activities cannot be ignored. In-depth studies of the sources and directions of agricultural N_2_O emissions will continue to be research hotspots and to present difficulties in the future.

## Figures and Tables

**Table 1:** Direct emission factors of agriculture induced N_2_O.

Region	N_2_O Direct Emission Factor (EF) (%)	Range (%)
Zone I (Inner Mongolia, Xinjiang, Gansu, Qinghai, Tibet, Shaanxi, Shanxi, Ningxia)	0.56	0.15~0.85
Zone II (Heilongjiang, Jilin, Liaoning)	1.14	0.21~2.58
Zone III (Beijing, Tianjin, Hebei, Shandong, Henan)	0.57	0.14~0.81
Zone IV (Zhejiang, Shanghai, Jiangsu, Anhui, Jiangxi, Hubei, Hunan, Sichuan, Chongqing)	1.09	0.26~2.20
Zone V (Guangdong, Guangxi, Hainan, Fujian)	1.78	0.46~2.28
Zone VI (Yunnan, Guizhou)	1.06	0.25~2.18

**Table 2:** Nitrous oxide emission parameters of main crops.

Crop	Nitrogen Content of Grain (%)	Nitrogen Content of Straw (%)	Economic Coefficient (%)	Root-to-Crown Ratio (%)	Proportion of Straw Returned to the Field (%)
Rice	0.0100	0.00753	0.489	0.125	32.30
Corn	0.0170	0.00580	0.438	0.170	9.30
Wheat	0.0140	0.00516	0.434	0.166	76.50
Sorghum	0.0170	0.00730	0.393	0.185	4.00
Soybean	0.0600	0.01810	0.425	0.130	9.30
Vegetables	0.0080	0.00800	0.830	0.250	61.85
Hemp	0.0131	0.01310	0.830	0.200	9.30
Potato	0.0040	0.01100	0.667	0.050	39.92
Tobacco	0.0410	0.01440	0.830	0.200	61.85
Sweetener	0.0055	0.00550	0.271	0.150	61.85

**Table 3:** Emission factors for livestock and poultry manure in different regions.

Region	North	Northeast	East	South Central	Southwest	Northwest
Cattle	0.794	0.913	0.846	0.805	0.691	0.545
Sheep	0.093	0.057	0.113	0.106	0.064	0.074
Goat	0.093	0.057	0.113	0.106	0.064	0.074
Pig	0.227	0.266	0.175	0.157	0.159	0.195
Poultry	0.007	0.007	0.007	0.007	0.007	0.007
Horse	0.33	0.33	0.33	0.33	0.33	0.33
Donkey	0.188	0.188	0.188	0.188	0.188	0.188
Mule	0.188	0.188	0.188	0.188	0.188	0.188
Camel	0.33	0.33	0.33	0.33	0.33	0.33

**Table 4:** Descriptive statistical analysis.

Variable Name	Minimum	Maximum
Yield of major crops (Ten thousand tons)	90,506	143,452
Number of livestock and poultry (Ten thousand units)	566,652	898,715
Amount of agricultural fertilizer application (Ten thousand tons)	4146	6023
Total output value of agriculture, forestry, animal husbandry and fishery (One hundred million yuan)	24,916	123,968
N_2_O emissions (Ten thousand tons)	66	81

## Data Availability

The data used to support the findings of this study are available from the corresponding author upon request.
